# Estrogen Induced Metastatic Modulators MMP-2 and MMP-9 Are Targets of 3,3′-Diindolylmethane in Thyroid Cancer

**DOI:** 10.1371/journal.pone.0015879

**Published:** 2011-01-18

**Authors:** Shilpi Rajoria, Robert Suriano, Andrea George, Arulkumaran Shanmugam, Stimson P. Schantz, Jan Geliebter, Raj K. Tiwari

**Affiliations:** 1 Department of Microbiology and Immunology, New York Medical College, Valhalla, New York, United States of America; 2 Department of Otolaryngology, New York Eye and Ear Infirmary, New York, New York, United States of America; Baylor College of Medicine, United States of America

## Abstract

**Background:**

Thyroid cancer is the most common endocrine related cancer with increasing incidences during the past five years. Current treatments for thyroid cancer, such as surgery or radioactive iodine therapy, often require patients to be on lifelong thyroid hormone replacement therapy and given the significant recurrence rates of thyroid cancer, new preventive modalities are needed. The present study investigates the property of a natural dietary compound found in cruciferous vegetables, 3,3′-diindolylmethane (DIM), to target the metastatic phenotype of thyroid cancer cells through a functional estrogen receptor.

**Methodology/Principal Findings:**

Thyroid cancer cell lines were treated with estrogen and/or DIM and subjected to *in vitro* adhesion, migration and invasion assays to investigate the anti-metastatic and anti-estrogenic effects of DIM. We observed that DIM inhibits estrogen mediated increase in thyroid cell migration, adhesion and invasion, which is also supported by ER-α downregulation (siRNA) studies. Western blot and zymography analyses provided direct evidence for this DIM mediated inhibition of E_2_ enhanced metastasis associated events by virtue of targeting essential proteolytic enzymes, namely MMP-2 and MMP-9.

**Conclusion/Significance:**

Our data reports for the first time that DIM displays anti-estrogenic like activity by inhibiting estradiol enhanced thyroid cancer cell proliferation and *in vitro* metastasis associated events, namely adhesion, migration and invasion. Most significantly, MMP-2 and MMP-9, which are known to promote and enhance metastasis, were determined to be targets of DIM. This anti-estrogen like property of DIM may lead to the development of a novel preventive and/or therapeutic dietary supplement for thyroid cancer patients by targeting progression of the disease.

## Introduction

The incidences of thyroid proliferative diseases (TPD) including thyroid cancer and goiter, are ever increasing with thyroid cancer being the most common among endocrine cancers [1], [2]. Recent statistics reveal 37,000 new cases were diagnosed in the US alone in 2009 and worldwide almost 27 million patients are affected [2]. The well differentiated papillary and follicular thyroid cancers variants account for more than 90% of all thyroid cancers and are invasive & metastatic [3]. Current treatments for TPD include surgery involving complete or partial removal of the thyroid gland, radioactive iodine (I^131^) therapy, chemotherapy or combination of all [4]. These treatments frequently require patients to take replacement thyroid hormones throughout life [4], [5] with the recurrence rate being unacceptably high, reaching almost 20–30% [5], [6]. In recent years, the recurrence rate and non-responsiveness to conventional thyroid treatments has increased thus warranting investigation of new preventive and therapeutic measures preferably using natural compounds present in diet.

Diet has always been of prime importance in its association with cancer development and prevention [7]–[9]. With respect to cancer prevention, several studies have found an inverse association of cancer risk with consumption of dietary products, such as tomatoes, soy and cruciferous vegetables [7]–[10]. In particular, cruciferous vegetables such as broccoli, kale, cauliflower and cabbage have been accepted by several organizations including the National Cancer Institute and Federal Drug Administration, to have a preventive effect against tumor development, especially in estrogen responsive tissue [7], [8]. Cruciferous vegetables contain several phyto-chemicals including Indole-3-carbinol (I3C), which is an effective oral chemopreventive agent against breast and prostate cancers [11]–[13]. I3C spontaneously converts to its dimeric product, 3,3′-diindolylmethane (DIM) at a low pH [11]–[14]. DIM is a stable compound and a safety evaluation reveals that long term administration of DIM (upto 12 months) in mice did not lead to any overt renal, cardio or gastro-intestinal toxicity [15]. This suggests that DIM may be a promising naturally available bioactive compound which can be used as an anticarcinogenic agent as it provides a safer and predictable response.

Currently, the precise cellular and biochemical mechanism by which DIM exerts its anticarcinogenic effects remains to be fully elucidated but based on available literature, DIM interferes with various signal transduction pathways. In breast and prostate cancers, DIM has been observed to induce dose dependent apoptosis by inhibiting AKT kinase and IKK-mediated IκBα phosphorylation, thus leading to inactivation of AKT and translocation of NFκB to the nucleus, resulting in decreased cell growth and proliferation [16], [17]. Also, it has been reported that DIM exerts its chemopreventive effects in hormone dependent cancers such as breast by upregulation of p21^WAFI/CIP1^ and the activation of the JNK pathway [18]. Interestingly, one potential target of DIM's activity is estrogen metabolism. Dalessandri et al. has shown that DIM increased the levels of 2-hydroxyestrones in postmenopausal women with a history of breast cancer resulting in an overall increase in 2-hydroxyestrones to 16α-hydroxyestrone ratio [19] thus favoring anti-proliferative conditions. Smith et al. have demonstrated that DIM, along with a phytoestrogen, Genistein can modulate estrogen metabolism towards 2-hydroxylation in estrogen sensitive prostate cancer cells [20]. Recently, we have also discovered in our laboratory that DIM can modulate estrogen metabolism in patients with TPD resulting in an increase in the ratio of 2-hydroxyestrones to 16α-hydroxyestrone (unpublished data) suggesting the use of dietary compounds, such as DIM, in TPD prevention.

Based on all the information available and discussed in the literature, this study was designed to define the mechanism(s) responsible for the anti-cancer effects of DIM in thyroid cancer. We report that DIM may have therapeutic effects by acting as a chemo-preventive agent possessing anti-estrogenic properties in thyroid cells by virtue of downregulating the estrogen induced cellular phenotypes of adhesion, invasion and migration. Our data indicates that the anti-estrogen like property of DIM is attributed to targeting the expression of matrix metalloproteinases (MMPs), which are essential proteases involved in adhesion, invasion and migration of cancer cells. Furthermore, the fact that MMPs are under the transcriptional regulation of the estrogen response element (ERE) lends further credence that DIM possesses anti-estrogenic properties.

## Materials and Methods

### Cell culture

Four thyroid cell lines were used in this study, BCPAP (human papillary thyroid cancer cell line), 8505C (human papillary thyroid cancer cell line), CGTHW-1 (human follicular thyroid cancer cell line) and ML-1 (human follicular thyroid cancer). BCPAP, 8505C and CGTHW-1 were cultured in RPMI-1640 (Mediatech, Herndon, VA) supplemented with 10% fetal bovine serum (FBS) (Atlanta Biologicals, Atlanta, GA), penicillin 10,000 IU/ml, streptomycin 10,000 µg/ml (Mediatech) and 2 mM L-glutamine (Mediatech) ML-1 was grown in DMEM (Mediatech) supplemented with 10% FBS, penicillin 10,000 IU/ml, streptomycin 10,000 µg/ml and 2 mM L-glutamine. BCPAP, 8505C, CGTHW-1 and ML-1 cell lines were purchased from DSMZ, Braunschweig, Germany. MCF-7 (human breast cancer cell line) was obtained from American Type Culture Collection (ATCC) (Manassas, VA) and grown in DMEM supplemented with 10% FBS, penicillin, streptomycin, insulin and L-glutamine.

### Western Blot analysis

Cells were harvested using 0.25% trypsin (Mediatech), washed with PBS, and lysed (1×10^6^/100 µL of lysis buffer) using the radioimmunoprecipitation assay (RIPA) buffer [50 mM Tris-HCl (pH 7.4), 150 mM NaCl, 0.2% sodium deoxycholate, 0.1% SDS, 0.5% NP40, 1 µM Pefabloc] and incubated on ice for 30 minutes with intermittent vortexing. The lysates were centrifuged at 14,000 rpm for 30 min at 4°C and supernatant were collected. Cell lysates (20 µg protein) were subjected to 12% SDS–PAGE under reducing conditions (presence of β-mercaptoethanol) as described earlier [21], [22]. Briefly, the proteins were transferred to Immobilon-P membranes at 220 mA for 2 h and membranes were blocked with 4% dried milk in TBST [200 mM Tris–HCl, pH 7.4, 150 mM NaCl, and 0.01% Tween-20 added fresh/liter of 1× TBS (TBST)] for at least 2–3 h on a shaker at room temperature. Subsequently, the membranes were incubated overnight at 4°C with either ER-α (Abcam, Cambridge, MA), ER-β (Santa Cruz Biotechnology, Santa Cruz, CA) antibody or actin (Santa Cruz Biotechnology) in TBST. Membranes were washed three times with TBST and incubated with the respective horseradish peroxidase (HRP) conjugated secondary antibody, for 2 hours at room temperature in TBST containing 2% milk. After four washes with TBS-T and one wash with TBS, membranes were developed by ECL substrate (Pierce Rockford, IL) and detected on Denville autoradiography films.

### XTT cell proliferation assay

Two thousand cells in 200 µl medium were plated into each well of 96-well plates and incubated overnight to allow cell adherence. The media was removed and 3,3′-diindolylmethane (DIM), kindly provided by Dr. Michael Zeligs (BioResponse, Boulder, Colorado) was added at concentrations of either 10, 25, 50, 75, 100, or 500 µM in a total volume of 200 µl and incubated for 24 h. The media were discarded and fresh growth media were added without DIM followed by addition of 50 µl XTT [1 mg/ml in serum-free RPMI + phenazine methosulfate (25 nM)]. After 4 hours of incubation, the OD was taken in a microplate reader at 450 nm and reference at 630 nm. The mean OD values were calculated for each dose at the respective time points and the percent survival as a function of time and dose was used to compare the experimental and control groups.

### Clonogenic Assay

To determine the effect of DIM on the clonogenicity, BCPAP, 8505C, CGTHW-1 and ML-1 cells were plated in six-well plates (200 cells per well). The cells were allowed to adhere overnight after which fresh media containing ±10^−8^ M E_2_ ±50 µM DIM was added or left untreated. After 21 days in culture, the cells were fixed and stained using 0.025% Coomassie brilliant blue R250 (in 50% methanol and 10% acetic acid) to visualize cell colonies. The colonies were counted, and percentage inhibition of clonogenicity was determined in the treated cells.

### Transwell Migration Assay

BD Biocoat Control Inserts (BD Biosciences, Bedford, MA) with 8-µm pore membrane filters were used for the migration assay as previously described [21], [23]. Briefly, cells were starved for 18 hours using starvation medium [phenol red-free medium supplemented with 10% charcoal stripped FBS (Sigma Chemical Co.) and penicillin (10,000 IU/ml)]. Cells were then harvested by trypsinization and 2.5×10^4^ cells were seeded in the upper chamber in 500 µl of media containing 1% FBS ±10^−8^ M E_2_ ±10^−6^ M fulvestrant ±25 µM DIM. The lower chamber contained 750 µl of media supplemented with 5% FBS. After 18 hours of incubation, the non migrating cells were removed from the upper surface of the membrane by gently scrubbing using cotton tipped swab. Cells on the lower surface of the membrane were then fixed using methanol and stained using 1% toluidine blue 1% borax stain followed by two washes with distilled water. Inserts were then allowed to airdry and counted in 10X field. Data are expressed as numbers of migrated cells per 10X field micrograph for each sample well and normalized to cell counts obtained from the untreated control.

### Scratch Wound Assay

Migratory ability of BCPAP, 8505C, CGTHW-1 and ML-1 cells was also assessed by a scratch wound assay. 5×10^5^ cells were plated in a six well plate and allowed to adhere and grow to 60–70% confluent cell monolayers. Subsequently, three vertical wounds were caused per well using a 2.5 µl sterile pipette tip followed by removal of any cellular debris and detached cells. The wounded cell monolayer was then incubated in fresh complete media with or without 25 and 50 µM DIM and estradiol. One horizontal line was made to allow visualization of cells at the same point. The cells were inspected every three hours until the scratch cells for control were fully migrated from one end of wound to other, which was 24 hours for BCPAP, 8505C and CGTHW-1 and 48 hours for ML-1. Pictures were taken just above and below the horizontal mark using a light microscope at 5X.

### Cell Adhesion assay

BCPAP, 8505C, CGTHW-1 and ML-1 cells were harvested as described and seeded at a density of 5×10^5^ cells per well in 6-well culture dishes in media supplemented with ±10^−^8 M E_2_ ±10^−6^ M fulvestrant ±25 µM DIM or left untreated and allowed to adhere for 2 hours. After indicated time points, medium with non-adhered cells was discarded and wells were gently washed twice with PBS to remove any loosely attached cells. Adherent cells were then scraped and counted using 0.4% trypan blue solution (Sigma Chemical Co., St. Louis, MO). The numbers of adherent cells were counted using a hemocytometer and the effect of DIM on cell adhesion was expressed as percent decrease in adherent cell count for cells treated with DIM relative to control cells.

### Invasion assay

Invasion assay was performed as previously described [21] using BD Biocoat growth Factor Reduced Matrigel Invasion chambers (BD Biosciences, Bedford, MA) with 8-µm pore membrane filters which were coated with matrigel. The protocol was essentially the same as migration assay except that growth factor reduced matrigel invasion chambers were rehydrated for 2 hours using serum free RPMI media at 37°C prior to loading cells onto inserts. Once rehydrated, 2.5×10^4^ cells were resuspended in the RPMI (500 µl) containing 1% FBS with ±10^−8^ M E_2_ ±10^−6^ M fulvestrant ±25 µM DIM and were carefully transferred onto the upper surface of filters in the chamber. Cells were allowed to invade for 18 hours after which cells were stained and counted similarly as described for migration assay. Percent invasion was calculated based on the percent of cells invading through the growth factor reduced matrigel invasion chambers relative to the cells migrating through control membrane.

### MMP detection

Thyroid cells were seeded at a density of 5×10^5^ cells per well in 6-well culture dishes and allowed to adhere overnight after which they were then switched to serum free medium and incubated with ±10^−8^ M E_2_ ±10^−6^ M fulvestrant ±25 µM DIM or left untreated for 24 hours. Conditioned medium was harvested, centrifuged to remove any debris, and aliquots were stored at −80°C until analyzed for MMP-2 and MMP-9 secretion and activity as described earlier [24], [25]. Briefly, the total protein concentration of the conditioned medium was determined using the Bio-Rad protein assay dye and equal proteins were used for each experiment. For gelatin zymography, SDS-PAGE gels were copolymerized with 0.1% gelatin (Sigma Chemical Co.) and 1 µg protein was resolved under non-reducing conditions. Following electrophoresis, gels were washed twice in renaturation buffer (2.5% Triton X-100 in distilled water) for 1 h on an orbital shaker. The zymograms were incubated for 1 h at room temperature followed by 36 hours at 37°C in zymography buffer (5 mM CaCl_2_, 0.2 mM NaN_3_ and 1 µM ZnCl_2_ in 50 mM Tris–HCl) with buffer changes every 12 hours. Gels were then stained with Coomassie blue (G-250 stain, Bio-rad Laboratories, Hercules, CA) and destained with 10% methanol and 7.5% acetic acid. Western blot analysis was performed using conditioned medium from thyroid cancer cells (equal protein concentrations) as described in the previous section using MMP-2 and MMP-9 (Cell Signaling Technology Inc., Danvers, MA) antibodies. For MMP inhibition, general MMP inhibitor 1,10 phenanthroline (Sigma Chemical Co.) was used. 2.5×10^4^ cells were resuspended in the RPMI (500 µl) containing 1% FBS with ±10^−8^ M E_2_ ±20 µM 1,10 phenanthroline ±25 µM DIM and seeded on BD Biocoat Control Inserts for migration and matrigel coated inserts for invasion as described in previous sections.

### siRNA and transfection conditions

For silencing estrogen receptor studies, ER-α small interfering RNA (siRNA) and siRNA transfection reagent (DharmaFECT1) were obtained from Dharmacon (Dharmacon, Inc., Lafayette, CO). BCPAP and MCF-7 cells were plated in 6 well plates and allowed to adhere overnight. Cells were transfected for 48 hours, and subsequently trasfected cells were harvested and seeded on BD Biocoat Control Inserts (migration) and matrigel coated inserts (invasion) in media containing 1% FBS ±10^−8^ M E_2_ ±10^−6^ M fulvestrant ±25 µM DIM. Non-transfected thyroid cancer cells treated with ±10^−8^ M E_2_ ±10^−6^ M fulvestrant ±25 µM DIM were used as appropriate positive controls. The migration and invasion studies were performed as described in previous sections.

### Statistical Calculation

Experiments presented here represent three replicates with statistical significance determined using a paired Student's *t* test with a probability (‘*p*’ value) ≤0.05 used to reject the null hypothesis.

## Results

### Thyroid cells express estrogen receptor

**Figure 1:**
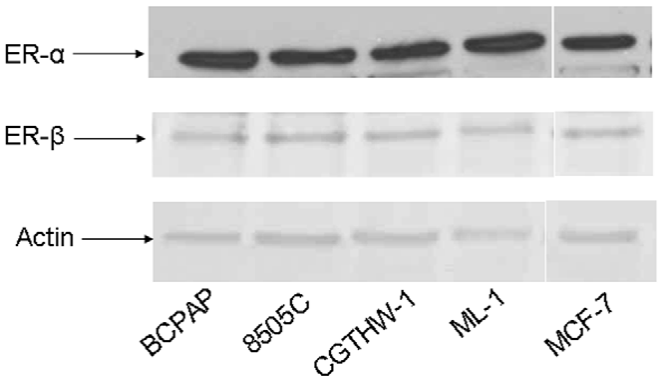
Thyroid cells express estrogen receptor. Whole cell protein (20 µg) was resolved by SDS-PAGE followed by Western blot analysis for ER-α (dilution 1∶500), ER-β (dilution 1∶1000) and actin (dilution 1∶5000). All the cell lines used in this study (BCPAP, 8505C, CGTHW-1 and ML-1) express both ER-α and ER-β at comparable levels.

The thyroid is not known to act as a traditional estrogen responsive tissue such as breast. In order to determine the status of the estrogen receptor in thyroid cells, we used four thyroid cell lines as our cell culture models. BCPAP and 8505C are human papillary thyroid cancer cell lines, CGTHW-1 and ML-1 are human follicular thyroid cancer cell lines. We observed that all the thyroid cell lines assayed expressed both isoforms of estrogen receptor (ER), ER-α and ER-β (Fig. 1) at comparable levels. MCF-7, a classical ER expressing breast cancer cell line, was used as a positive control for detection of both polymorphic forms of ERs (ER-α and ER-β).

### DIM has anti-proliferative effect on thyroid cells

DIM, a natural compound from cruciferous vegetables has been observed to have anti-proliferative properties against several hormone responsive cancers [14], [16]–[18]. We wanted to evaluate the effect of DIM on the proliferative activity of thyroid cancer and in order to do so, an XTT assay was performed and the results are shown in Table 1. All the cell lines (BCPAP, 8505C, CGTHW-1 and ML-1) used in this study were treated with various concentrations of DIM for 24 hours. A dose dependent inhibition in cell viability was observed with a 50% inhibition at a concentration of approximately 50 µM DIM during 24 h of treatment in all four cell lines, BCPAP, 8505C, CGTHW-1 and ML-1. Based on these observations (Table 1), the 25 µM DIM concentration was used to further characterize the effects of DIM on thyroid cells at both the molecular and phenotypic levels.

**Table 1:** Thyroid cell survival assay.

DIM concentration (µM)	BCPAP	8505C	CGTHW-1	ML-1	MCF-7
untreated	100±0	100±0	100±0	100±0	100±0
10	91.9±9	98.1±2.9	95.1±4.2	90.8±0.9	86.4±0.1
25	87.9±7.5	88.9±0.8	85.8±7.4	77.7±5.6	62.6±0.3
50	43.8±4.3	49.0±5.4	46.4±4.4	54.2±3.9	46.5±2.6
100	18.6±8.9	28.8±3.4	14.5±2.9	31.6±4.6	23.4±4.8
500	1.19±0.6	21.9±7.2	4.14±2.5	19.1±3.7	21.2±0.9

The numbers above represent the percent of viable cells with various concentrations of DIM. The percent survival was calculated based on untreated cells which were set as 100%.

### DIM inhibits the clonogenic ability of thyroid cells

One of the hallmark properties of cancer cells is the ability to divide under isolated conditions using minimal paracrine and possibly more autocrine factors [26]. The anticancer effect of DIM on the ability of BCPAP, 8505C, CGTHW-1 and ML-1 cells to divide indefinitely was evaluated by a clonogenic cell survival assay. Two hundred cells per well in six well plates were treated with 50 µM DIM ± estradiol for 21 days. As shown in Table 2, the untreated thyroid cells have the ability to form clones whereas estrogen treatment resulted in approximately 8 (8505C) −33% (ML-1) increase in clone formation depending on cell lines. DIM completely abrogated clone formation of all cell lines irrespective of whether they were treated with estradiol. These experiments establish the differential responsiveness of different thyroid cancer cells to estradiol and the anti-estrogenic activity of DIM.

**Table 2:** DIM inhibits clonogenicity of thyroid cells.

Treatments	BCPAP	8505C	CGTHW-1	ML-1	MCF-7
untreated	100±0	100±0	100±0	100±0	100±0
10^−8^ M E_2_	109±2.3	108.1±10.7	122.5±4.3	133.3±1.4	154.7±8.7
50 µM DIM	0	0	0	0	0
10^−8^ M E_2_+50 µM DIM	0	0	0	0	0

Thyroid cell were cultured under various conditions for 21 days. The numbers above are in % clone and were calculated based on untreated cells which were set as 100%.

### DIM reduces the migratory ability of thyroid cells

**Figure 2:**
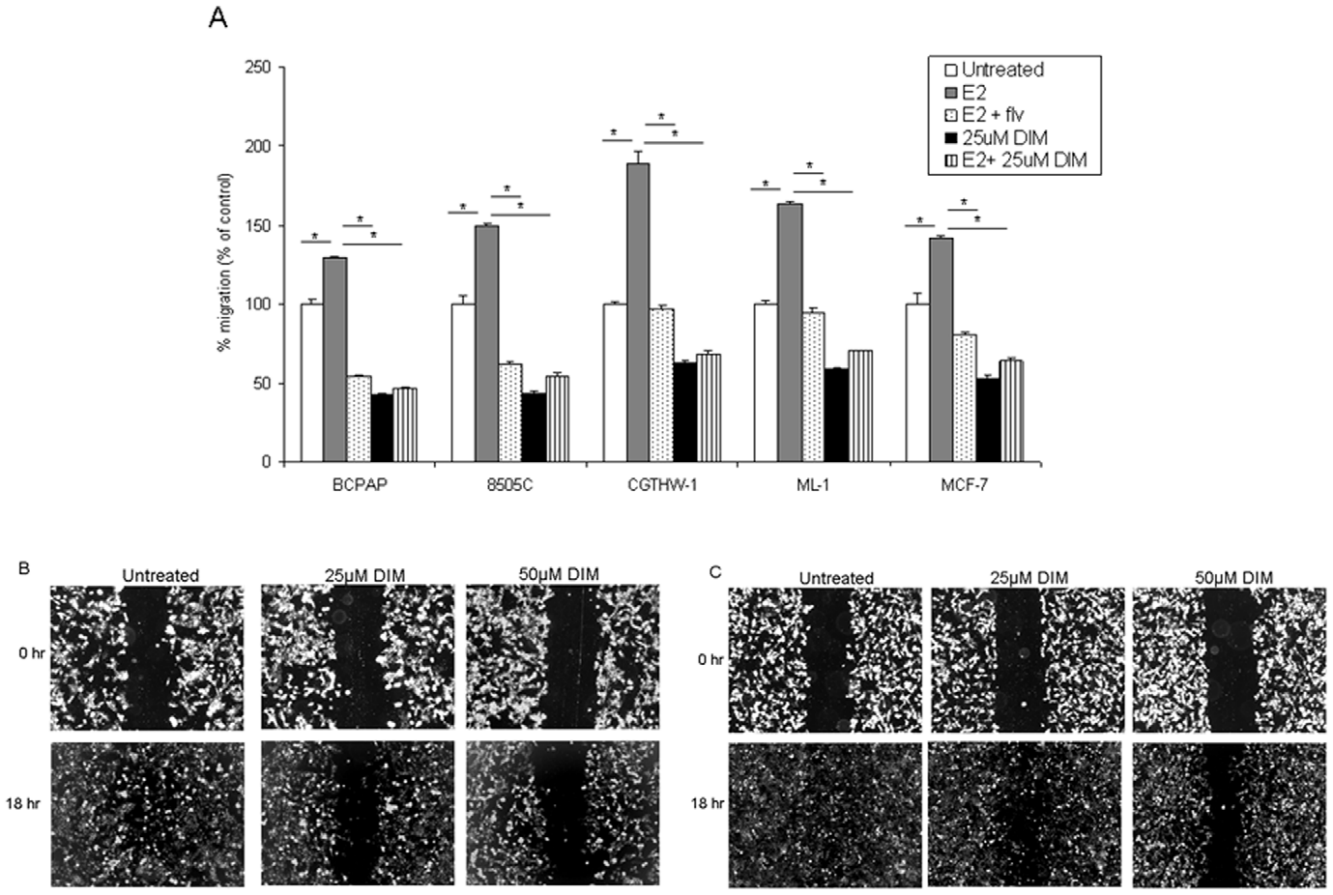
DIM reduces migration of thyroid cells. (A) 2.5×10^4^ cells were resuspended in 500 µl of RPMI with 1% FBS ±10^−8^ M E_2_ ±10^−6^ M fulvestrant ±25 µM DIM and seeded in the upper chamber of BD Biocoat Control Inserts (8-µm pore membrane filters). 750 µl of RPMI containing 5% FBS was added to the bottom chamber as a chemoattractant. After 18 hours, the cells that migrated were fixed, stained, and counted under the 10X objective. The groups are as follows- untreated (white bars), E_2_ treated (grey bars), E_2_ + fulvestrant (dotted bars), 25 µM DIM (black bars) and E_2_ +25 µM DIM treated (striped bars). Data is expressed as numbers of cells counted (migrated cells) for each sample and normalized to the cell number obtained from the untreated control. The asterisk denotes statistically significant differences (*p*<0.05) between the indicated samples. Scratch wound assay for BCPAP (B) and CGTHW-1 (C). 5×10^5^ cells were plated and allowed to grow to semi confluent monolayers after which a ‘vertical wound’ was created and cells were then allowed to migrate in the presence of either 25 µM or 50 µM DIM. The cells were visualized under 5X every three hours and photographic documentation was taken at 18 hours when the cells completely migrated from one end of ‘scratch’ to other end in untreated controls.

Tumor cells have an enhanced ability to migrate into neighboring tissue and certain agents such as estradiol have been observed to aid in this migration process. Therapeutic agents which can lower the ability of these cells to migrate can presumably effectively lower the risk of metastasis. We have previously demonstrated that thyroid cells have the ability to increase migration in response to estrogen [21] and to determine if DIM can affect the migratory potential of these cells, *in vitro* migration assays were performed. Starved thyroid cells were seeded in a transwell migration chamber with estradiol ± fulvestrant or ±25 µM DIM and allowed to migrate. We observed that thyroid cells were capable of migrating and this ability to migrate was enhanced when cells were treated with E_2_ (grey bars) compared to untreated cells with 30% increase in migration for BCPAP, 50% for 8505C, 89% for CGTHW-1 and 63% for ML-1 (Fig. 2A). This increase in cell migration was abrogated when fulvestrant was used along with estradiol [dotted bars) suggesting that the migratory ability of these thyroid cells is influenced by estrogen and an estrogen receptor antagonist has abrogated the migration of these cells. DIM (25 µM) significantly decreased migration of thyroid cells (black bars). This decrease was observed to be approximately 37–57% and was cell line dependent. Moreover, the addition of estradiol along with 25 µM DIM (striped bars) did not improve the migratory ability of thyroid cells, signifying the antiestrogen like ability of DIM.

To further validate the effect of DIM on the migratory capability of thyroid cells, a scratch wound assay was performed, which is a partial *in vivo* representation of metastatic phenotype [27]. Thyroid cells (BCPAP, 8505C, CGTHW-1 and ML-1) were grown in a 60–70% confluent monolayer followed by formation of scratches and subsequent incubation with ± DIM. We found that the DIM caused a decrease in cell migration by 50–60% (as observed visually) in a dose dependent manner in thyroid cells [BCPAP (Fig. 2B) and CGTHW-1 (Fig. 2C)]. Similar results were observed with 8505C and ML-1 (data not shown). We have also previously observed that estradiol enhances migration and this migration was inhibited by fulvestrant, similar to the scratch wound assay results obtained in this study using DIM [21].

### DIM decreases the adhesion ability of thyroid cells

**Figure 3:**
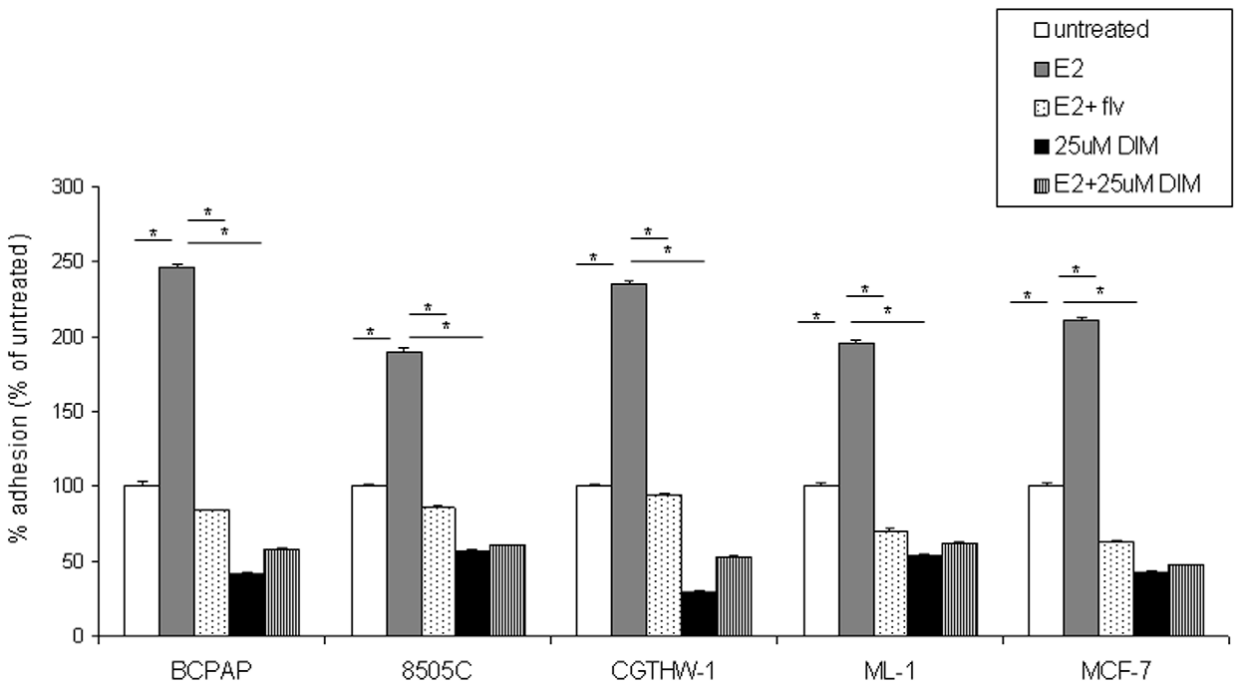
DIM decreases adhesion of thyroid cells. 5×10^5^ cells were suspended in complete medium containing ±10^−8^ M E_2_ ±10^−6^ M fulvestrant ±25 µM DIM and plated in 6 well culture dishes. After 2 hours viable adhered cells were removed by scraping and counted by trypan blue. The groups are as follows- untreated (white bars), E_2_ treated (grey bars), E_2_ + fulvestrant (dotted bars), 25 µM DIM (black bars), and E_2_ +25 µM DIM treated (striped bars). Data are expressed as % adhered cells compared to untreated cells which were set to 100% adhesion. Single asterisk denotes statistically significant differences (*p*<0.05) between the indicated samples.

In order to successfully metastasize, tumor cells have to adhere to the extra cellular matrix (ECM) of secondary organs [26], [28]. We evaluated the effect of DIM on adhesion of thyroid cells by performing an adhesion assay. Thyroid cells were allowed to adhere in the presence of 10^−8^ M E_2_ or ±10^−6^ M fulvestrant (a classical estrogen receptor antagonist) ±25 µM DIM for 2 hours and % adhesion was calculated (Fig. 3). An increase in adhesion was observed when cells were treated with E_2_ (grey bars), which was abrogated with fulvestrant (dotted bars) suggesting that adhesion is an active phenotype in thyroid cells possibly requiring functional ER activity. Interestingly, a significant decrease in adhesion was observed when cells were treated with 25 µM DIM (black bars), with a 58% decrease in adhesion for BCPAP, 42% for 8505C, 70% for CGTHW-1 and 45% for ML-1. Combination of estradiol and 25 µM DIM (striped bars) could not improve the adhesion of thyroid cells, suggesting that DIM may prevent estrogen enhanced adhesion and can potentially act as an effective anti-estrogen/metastatic agent, as the decrease in cell adhesion by DIM is similar to the decrease in adhesion observed with the estrogen receptor antagonist fulvestrant.

### DIM inhibits the invasion of thyroid cells

**Figure 4:**
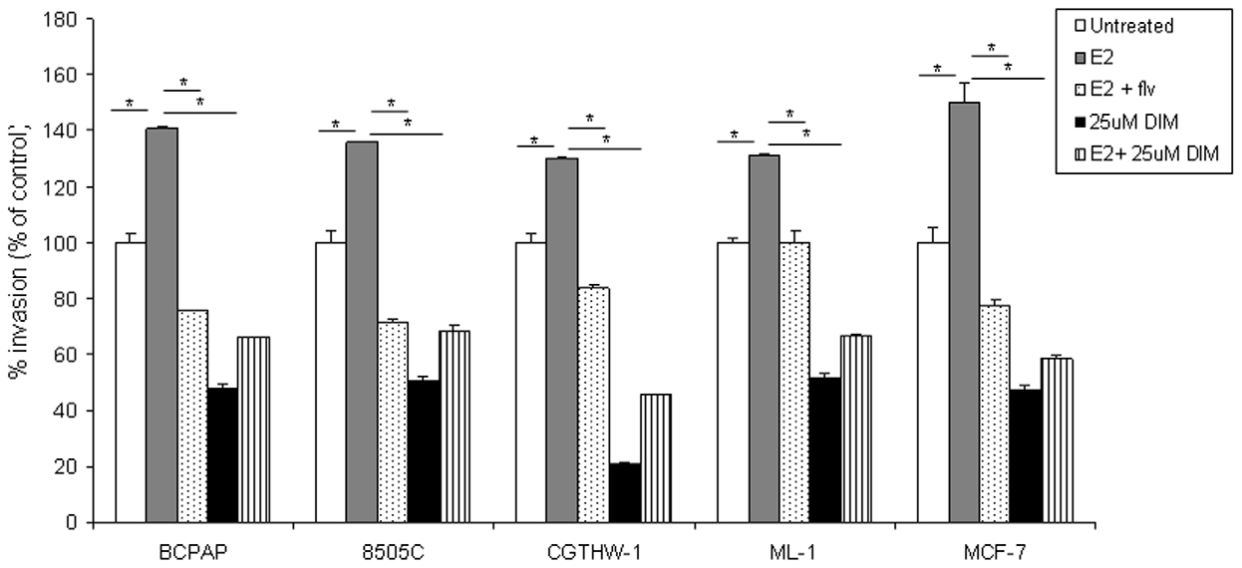
DIM reduces invasion of thyroid cells. 2.5×10^4^ cells resuspended in 500 µl medium containing 1% FBS ±10^−8^ M E_2_±10^−6^ M fulvestrant ±25 µM DIM were seeded in the upper chamber of growth factor reduced matrigel invasion chambers. 750 µl of growth medium containing 5% FBS was used as chemoattractant in the bottom chamber. Percent invasion was calculated based on the number of cells invading through the chambers relative to the cells migrating through control membrane after 18 hours. The groups are as follows- untreated (white bars), E_2_ treated (grey bars), E_2_ and ICI (dotted bars), 25 µM DIM (black bars) and E_2_ & 25 µM DIM treated (striped bars). Data is represented as % invasion which is mean number of invaded cells per 10X field micrograph for each sample well relative to the migration through the control membrane and normalized to the untreated control. The asterisk denotes statistically significant differences (*p*<0.05) between the indicated samples.

Formation of secondary metastatic foci by tumor cells requires invading the ECM of secondary organs [26] and inhibiting this invasion is a strategy in cancer therapy that has been widely investigated. The effect of DIM on the invasive potential of BCPAP, 8505C, CGTHW-1 and ML-1 was assayed through the use of a transwell invasion chamber coated with a biological matrix *in vitro* (matrigel). Thyroid cells were loaded in a transwell chamber with estradiol ± fulvestrant or ±25 µM DIM and were allowed to invade through the matrigel. We observed that thyroid cells were capable of invading through matrigel and invasion by these cells was enhanced when cells were treated with E_2_ (grey bars) with an 40% increase in invasion for BCPAP, 36% for 8505C, 30% for CGTHW-1 and 31% for ML-1 (Fig. 4). This increase in cell invasion was abrogated when fulvestrant was used in combination with estradiol (dotted bars) suggesting that an estrogen receptor antagonist can block the invasive propensity of thyroid cells. To evaluate the effect of DIM on invasion of thyroid cells, all the cell lines were treated with 25 µM DIM (black bars) and a significant decrease (*p*<0.05) in invasion was observed. This decrease in invasion was approximately 52% for BCPAP, 50% for 8505C, 80% for CGTHW-1 and 48% for ML-1 when compared to control cells (normalized to 100% and *p*<0.05). Furthermore, pro-proliferative estradiol, in combination with DIM (striped bars), did not improve the invasive ability of thyroid cells. This data suggests that DIM abrogates estrogen enhanced cellular processes of invasion thus possibly targeting a major step in tumor progression and metastasis.

### DIM suppresses estrogen induced MMP secretion

**Figure 5:**
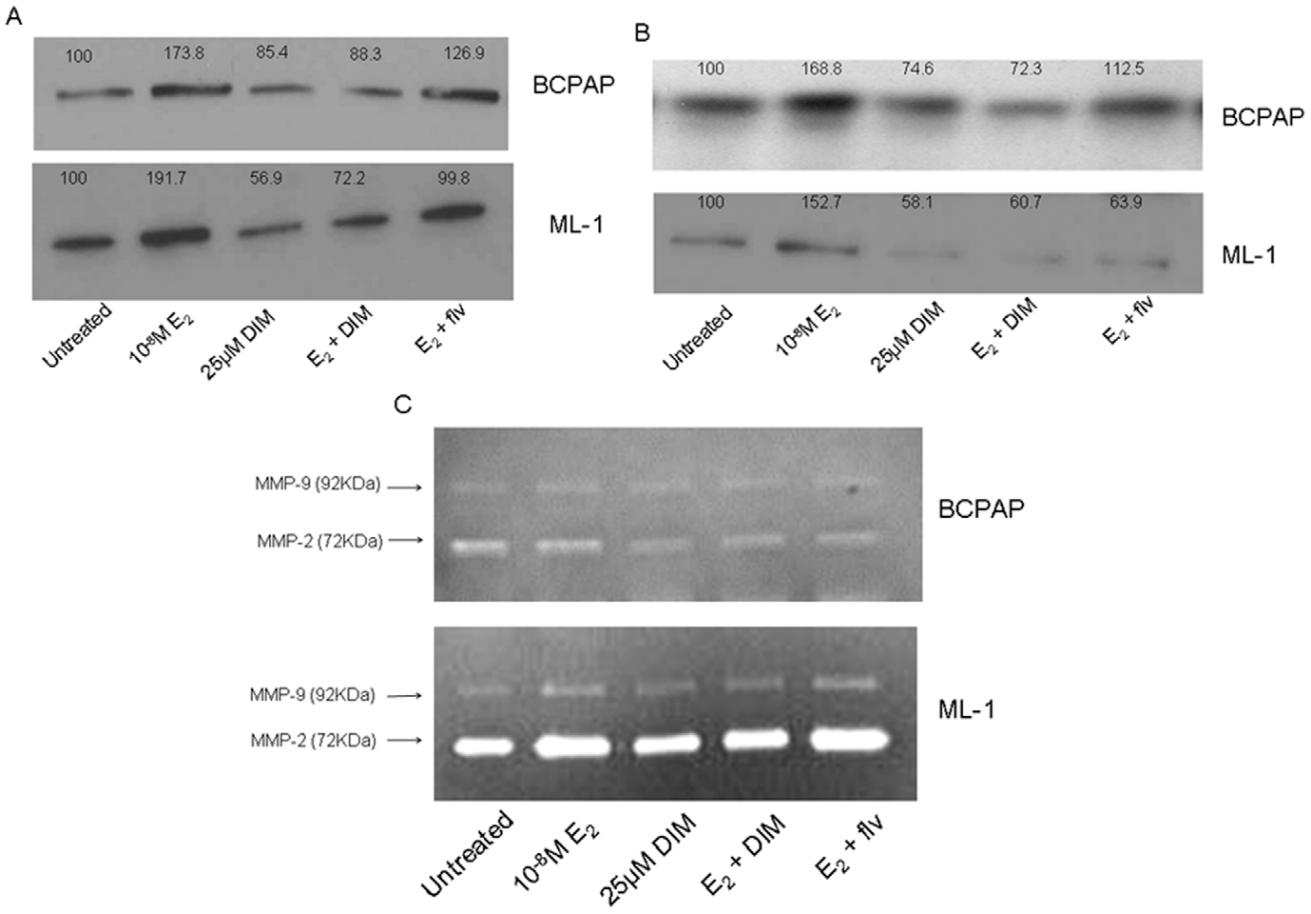
DIM reduces estrogen mediated enhancement of MMP-2/9 protein secretion and activity in thyroid cells. Conditioned medium from thyroid cells treated with 10^−8^ M E_2_±10^−6^ M fulvestrant ±25 µM DIM for 24 hours were collected and total protein concentration was determined. One microgram (µg) of protein for each treatment was resolved by SDS-PAGE followed by Western blot analysis for MMP-2 (dilution 1∶1000) (A), MMP-9 (dilution 1∶1000) (B) and zymography (C). In zymography, areas of MMP enzymatic activity appeared as clear bands over the dark blue background. Shown are densitometric values with untreated samples set as 100% and average of three independent experiments.

**Figure 6:**
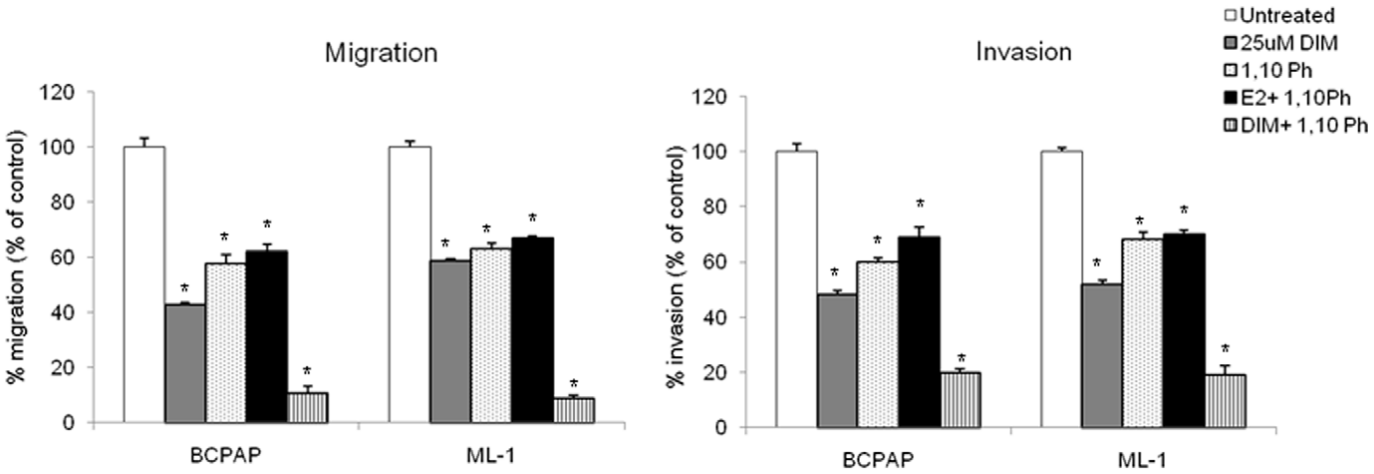
MMP inhibitor 1,10 phenanthroline attenuates migration and invasion of thyroid cells. Thyroid cells were treated with 10^−8^ M E_2_ ± MMP inhibitor (1,10 Ph) ±25 µM DIM or left untreated and percent migration and invasion of treated cells was determined compared to untreated cells which were set to 100%.

MMPs are reliable markers of tumor cell invasion and migration. Moreover, malignant tumors are known to have increased MMP expressions compared to benign tumors, which causes degradation of the extracellular matrix, resulting in increased invasion and migration of tumor cells [29], [30]. Phytochemicals such as genistein and I3C have been shown to target the activity and secretion of MMPs in estrogen responsive cancers [31]. Thus, to determine if estrogen and/or DIM, a phytochemical, could modulate MMP-2 and MMP-9 secretion and activity in thyroid cancer cells, we stimulated BCPAP and ML-1 cells with estrogen ±25 µM DIM or ± fulvestrant and measured MMP-2 and MMP-9 protein secretion and activity by performing Western blot analysis and zymography, respectively using conditioned medium from thyroid cancer cells. As seen in Fig 5, estrogen treatment resulted in an increased MMP-2 and MMP-9 protein secretion (5A & 5B) and activity (5C) compared to untreated controls. The levels of MMP secretion and activity were downregulated when thyroid cells were treated with 25 µM DIM. DIM was also able to suppress the estrogen induced enhanced secretion of MMP-2 as well as MMP-9 suggestive of its anti-estrogen like ability. To further evaluate the role of MMPs in thyroid cell invasion and migration, a general MMP inhibitor, 1,10 phenanthroline was used. 1,10 phenanthroline was able to significantly attenuate the migration and invasion of thyroid cells. Even when cell were subjected to 1,10 phenanthroline and estrogen (Fig. 6) together, the pro-migratory and invasive abilities of estrogen were nullified. More interestingly, when DIM and 1,10 phenanthroline were added together, only 9–11% of cells were able to migrate and 19–20% of cells invaded, suggesting a definitive role of these enzymes in the migration and invasion of thyroid cancer cells.

### ERα silencing attenuates DIM induced inhibition of Migration and Invasion

**Figure 7:**
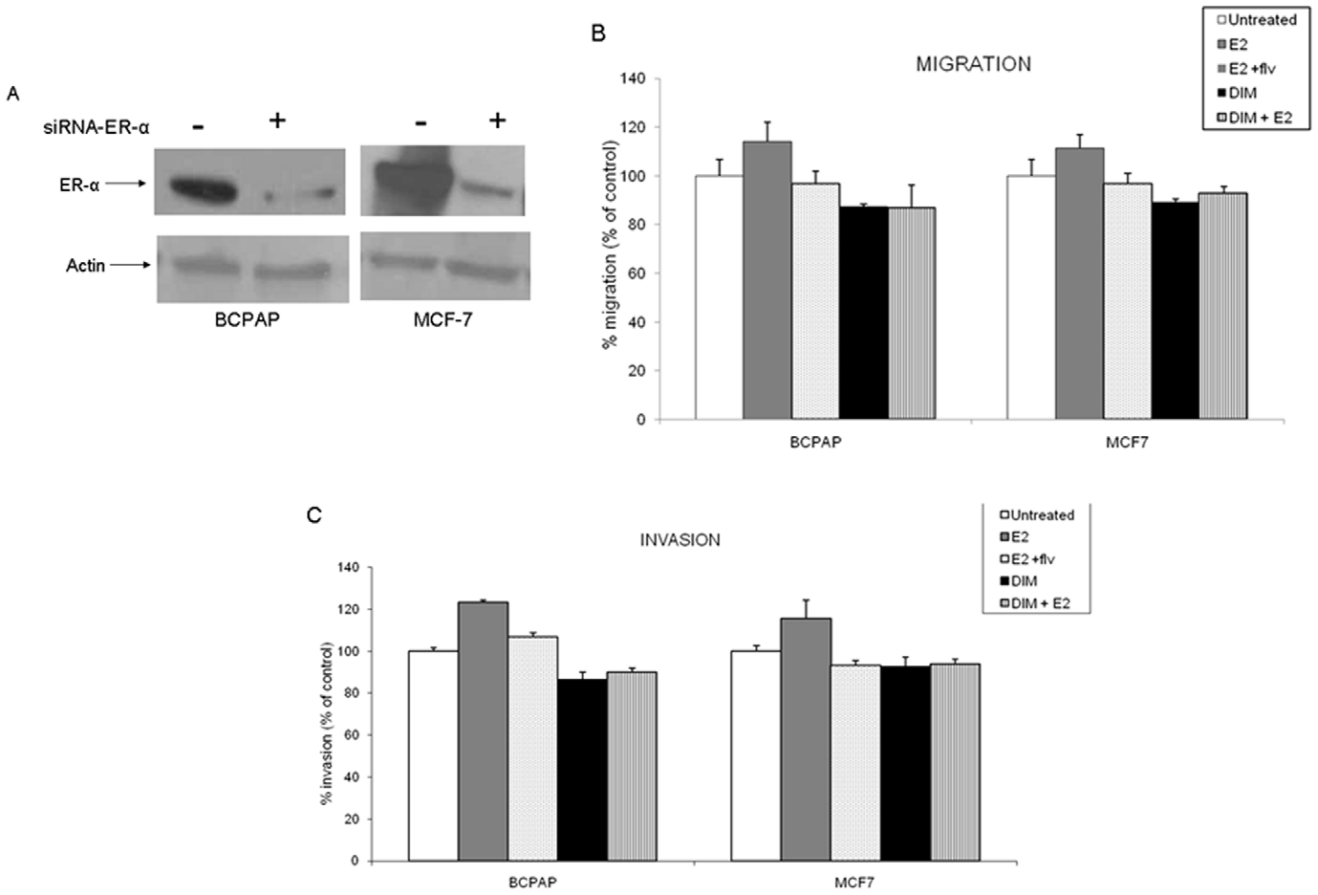
ER-α silencing inhibits DIM's effect on migration/invasion. (A) Western Blot analysis demonstrating downregulation of ER-α. BCPAP and MCF-7 cells were transfected with siRNA and whole cell protein was resolved by SDS-PAGE followed by Western blot analysis for ER-α. A decrease in ER-α expression was observed for both BCPAP and MCF-7 confirming ER-α silencing. Actin was used as a loading control. (B & C) Cancer cells were transfected with ER-α siRNA and subsequently treated with 10^−8^ M E_2_±10^−6^ M fulvestrant ±25 µM DIM or left untreated. Data presented demonstrates percent migration (B) and invasion (C) of ER-α silenced cancer cells treated with E_2_± fulvestrant ± DIM compared to untreated ER-α silenced cancer cells which were set to 100%. MCF-7 was used as positive control for ER silencing experiments.

Activation and secretion of MMP by estrogen is known to be ER dependent and this stimulation can lead to enhanced migration and invasion of tumor cells, thus, we used ER silencing to further elucidate the mechanism of action of estrogen and DIM on the migration and invasion of thyroid cells. The efficiency of ER silencing in BCPAP cells was determined by Western Blot analysis in which a decreased expression of ER was observed (Fig 7A). Migration and invasion were observed in untreated transfected cells suggesting that the process of transfection did not alter any cellular phenotype pertaining to the migratory and invasive properties of BCPAP. Loss of the estrogen receptor by siRNA tranfection resulted in loss of estradiol mediated migration and invasion of thyroid cancer cells with the activity of DIM also nullified (Fig 7B & 6C). The E_2_ stimulation on migration and invasion was minimal with 13% on migration and 23% on invasion for B-CPAP and the addition of DIM did not affect migration and invasion as the results were comparable to the control (Fig 7). As a further positive control for our ER silencing experiments, the human estrogen responsive breast cancer cell line, MCF-7, was used. The effectiveness of ER silencing for MCF-7 was also determined through Western Blot (Fig. 7A) and the migration and invasion results observed with MCF-7 (Fig 7B & 7C) were comparable to BCPAP. These results validate the essential function of estradiol in thyroid cancer cell metastasis and the target of DIM as being anti-estrogenic. These results were similar when the ER antagonist fulvestrant was added in the E_2_ stimulated transfected cells, further suggesting that the E_2_-ER signaling has significance in migration/invasion upon estrogen stimulation.

## Discussion

Thyroid cancer, the most prevalent endocrine-based cancer, occurs four to five times higher in women than in men suggestive of the possible involvement of estrogen in its development. We have recently demonstrated that thyroid cells are estrogen responsive and estrogen can not only modulate thyroid cancer cell proliferation but also metastasis associated events [21]. Estrogen mediated changes at the cellular level are mostly mediated via its receptors, ER-α and ER-β, which influence several pro-survival pathways [32], [33]. Estrogen is known to influence cell proliferation by inducing transcription of several genes such as c-jun, c-myc and c-fos, as well as growth factors, such as TGF-α and EGF and also directly influences cyclins that regulate the cell cycle [34]. Furthermore, the binding of estrogen to either membrane bound and/or cytosolic estrogen receptor results in activation of several intracellular signaling pathways, such as the PI3K/Akt and ERK pathways, which upon activation lead to anti-apoptotic signals [32]–[35]. Many reports indicate that these E_2_-ER mediated signals can subsequently result in development of the metastatic phenotype, characterized by increased cell proliferation, migration and invasion of several cancers, in particular, breast cancer [36]–[38]. The link between estrogen and thyroid cancer opens up the possibility of using anti-estrogens for thyroid cancer treatment.

Several anti-estrogenic synthetic compounds exist, such as tamoxifen and ICI 182,780 (fulvestrant), which prevent activation of the Akt and ERK pathways by virtue of their competition with E_2_ for binding to the ER [39]. Unfortunately, the use of these antiestrogens have undesirable side effects such as ICI 182,780 resulting in a complete blockade of activation pathways of ER and tamoxifen resulting in detrimental uterotrophic effects and can act as agonist for breast cancer cell growth [39], [40]. Overall, these damaging effects warrant the research for safer alternatives to synthetic antiestrogens. Interestingly, one way to possibly treat thyroid cancer is through the use of natural dietary compounds such as DIM, provided that DIM has been shown to effect estrogen responsive tissues such as breast.

DIM is a natural compound that is found in cruciferous vegetables and has widely been investigated for its anti-carcinogenic effects against several types of cancers such as breast and prostate [16], [17]. Although the exact mechanism of DIM's anti-carcinogenic property needs further investigation, several studies seem to concur that the downstream effects of DIM are to target essential events involved in cancer cell proliferation and metastasis. Our group has also observed these anti-proliferative actions of DIM but we are also ascribing a potentially new function to DIM, which is acting as an anti-estrogen by possibly targeting both genomic and non-genomic E_2_-ER signaling pathways. In this study, we observed that DIM acts in a similar fashion to the anti-estrogenic compound fulvesterant by inhibiting estrogen induced proliferation and clone formation of thyroid cancer cells. DIM was also observed to interfere with essential events involved in cancer cell metastasis as evidenced by a decrease in *in vitro* cell adhesion, migration, and invasion. Of these three crucial steps involved in initiating metastasis, invasion is the most essential one. An agent that could efficiently inhibit the ability of cancer cells to form secondary metastatic foci would be an ideal candidate to suppress cancer progression. We observed that DIM has a unique anti-invasive property, with as high as 78% inhibition of invasion observed in papillary thyroid cancer cells. Most importantly, we observed that estradiol mediated migration and invasion of thyroid cells is dramatically abrogated by DIM when DIM and estrogen were added together in cell cultures.

Tumor cell invasion is facilitated by proteolytic enzymes which degrade the extracellular matrix (ECM) of the surrounding tissue leading to the formation of secondary foci. Two such proteolytic enzymes are MMP-2 and MMP-9, which belong to a large family of zinc dependent endopeptidases involved in degradation of ECM proteins. MMPs are secreted by cells as proenzymes which become catalytically active by either autoactivation or by other proteinases. The ECM is not only a solid state support for cells, but it also serves as a reservoir for various essential bioactive molecules such as cytokines and growth factors which are released by MMP induced degradation [41] thus providing a depot of bioactive molecules that aid in tumor cell metastasis and angiogenesis. Interestingly, growth factors are demonstrated to stimulate MMP-9 activation in head and neck squamous cell carcinoma [42] and elevated levels of MMPs have been demonstrated in thyroid carcinoma. Yeh et. al demonstrated that MMPs are critical effectors of invasion in papillary and follicular thyroid cancer cell lines [43]. In addition, estrogen has been shown to regulate the activity of MMP-2 and MMP-9 in ER+ breast cancer cells [44] and a positive correlation between ER-α expression and the effects of estrogen on MMP gene expression in breast cancer cells [45]. In this study, we observed that MMP-2 and MMP-9 secretion was increased with estrogen treatment of ER+ thyroid cancer cells, which was suppressed by treatment with DIM, providing not only a correlative link but a direct validation of the anti-estrogenic activity of DIM that alters thyroid cancer cell phenotype. Therefore, our observations along with the aforementioned studies [44], [45] provide a strong correlative link between E_2_-ER signaling and MMP secretion. In addition, it is known from recent reports that the interactions between the cancer cells and stromal cells may enhance the growth and progression of tumor [46]. The exact mechanism by which DIM and/or estrogen are affecting the tumor microenvironment need to be further elucidated.

Lastly, recent publications which implicate the involvement of estrogen with increased metastatic phenotypic properties coupled with an observation in which DIM inhibits these estrogen mediated metastasis associated events (current study), prompted us to validate our observations by ER-knockdown studies using BCPAP. Silencing of the ER was able to abrogate the pronounced DIM mediated inhibition of E_2_ induced cell migration/invasion in normal non-transfected cells. This observed phenomenon was comparable to the ER silenced MCF-7 cell line thus authenticating the presumed antiestrogenic activity of DIM which appears to utilize the estrogen receptor and its ability to modulate E_2_-ER system and signal transduction pathways which are E_2_ mediated such as the pAkt and ERK pathways, as described earlier by us and others [14], [47]–[50].

Collectively, our observations provide evidence that DIM inhibits the estrogen mediated increase in thyroid cell migration, adhesion and invasion which was supported by ER-α downregulation studies. Lastly, we have identified a mechanism behind this estrogen induced migration, adhesion and invasion in thyroid cells as induction and activation of essential proteolytic enzymes, mainly MMP-2 and MMP-9. Taken together, our observations provide for the first time, direct evidence for the estrogen/ER mediated regulation of MMP secretion and activity in thyroid cancer cells. Overall, these findings open a new avenue and clinical utility for DIM as the prototypical anti-estrogen that can be used for therapeutic and preventive purposes of thyroid proliferative diseases by not only suppressing the proliferation of thyroid cancer cells but also by inhibiting metastasis associated events.
